# A Longitudinal Mixed-Methods Characterization of Family Support from Adolescence to Young Adulthood in Autism and Other Developmental Disabilities

**DOI:** 10.1007/s10803-023-06070-y

**Published:** 2023-09-05

**Authors:** Hillary K. Schiltz, Elaine Clarke, Nicole Rosen, Sofi Gomez De La Rosa, Nina Masjedi, Kourtney Christopher, Catherine Lord

**Affiliations:** Semel Institute for Neuroscience and Human Behavior, University of California, Los Angeles, 760 Westwood Plaza, Los Angeles, CA 90024 USA

**Keywords:** Autism, Adulthood, Family, Support

## Abstract

Although caregiving responsibilities and need for support persist and evolve across the life course in families with autistic youth or youth with other developmental disabilities (DDs), little is known about support during their child’s adulthood years. Therefore, the present study used a mixed-methods approach to examine change and stability in formal and informal family support across the transition to adulthood. Caregivers of 126 individuals with autism or DDs completed a modified version of the Family Support Scale, including open-ended questions, at five time points from adolescence (age 16) into young adulthood (age 22). Caregivers reported that informal support from family members was the most frequently used, helpful, and valued source of support with relative stability across time. In contrast, the reported helpfulness, use, and value of formal support (e.g., professionals, schools) for caregivers declined over time. Qualitative content analyses revealed characteristics of highly valued support included support type (e.g., instrumental or emotional) and features of the support source (e.g., their understanding). There was a shift to valuing emotional support more than instrumental support over time, especially for caregivers of less able adults. Partnership and dependability emerged as highly valued features of the support source. These findings fit within a social convoy perspective and likely reflect the “service cliff” experienced by autistic individuals or people with DDs and their families. As social networks shrink over time and formal services are less readily available in adulthood, remaining sources of support, particularly from family members, become increasingly important.

A strong family support system can promote positive parent (Angley et al., 2015; Taylor, 2011) and child outcomes (Taylor et al., 2015) and can bolster family functioning (Armstrong et al., 2005). For caregivers of autistic individuals and people with other DDs, family support is a particularly important resource and source of resilience (Peer & Hillman, 2014); it can buffer parenting stress and burden (Boyd, 2002; Kyzar et al., 2012; Robinson & Weiss, 2020; Robinson et al., 2016; Weiss et al., 2021), lead to decreased child behavior problems (Weiss et al., 2021), and promote caregiver physical health (Gouin et al., 2016), well-being (Benson, 2012; Ekas et al., 2010; Smith et al., 2012a) and quality of life (Kyzar et al., 2012; Vasilopoulou & Nisbet, 2016). Although it is clear that family support is beneficial for these families, much of the existing work on this topic is cross-sectional, conceptualizes support as unidimensional, and focuses on childhood and/or the period immediately following autism diagnosis (McGrew & Keyes, 2014; Zuna et al., 2016). Caregiving challenges evolve and often persist across the lifespan for families of youth with autism and DDs, with unique challenges in the years immediately preceding and following the transition to adulthood (Burke et al., 2018). Adulthood is a longer period than childhood with greater caregiving financial costs (Buescher et al., 2014; Cakir et al., 2020; Parish et al., 2004). Up to 80% of caregivers of individuals with autism and DDs continue to perform caregiving duties in adulthood (Heller et al., 2007; Shattuck et al., 2012, 2020). These findings underscore the importance of understanding the functions and changes in family support in this aging caregiver population.

Family support can be conceptualized as a multidimensional construct (Bromley et al., 2004; Marsack-Topolewski, 2020). Sources of support vary widely in both formality—from government-funded respite care to lunch with a friend—and in nature—from practical help with daily care tasks to providing a listening ear. Formal supports are usually financially compensated services, often by the family and/or state or federal channels. These can include adult day programs, vocational training, school-based services, and medical care. In contrast, informal supports are unpaid services typically provided by members of caregivers’ social networks, such as immediate and extended family members, friends, and neighbors (Smith et al., 2012a). Regarding the nature of family support, instrumental support includes assistance with practical caregiving tasks such as help with transportation, childcare, or household chores; emotional support encompasses empathy, kindness, and making someone feel loved and cared for (Morelli et al., 2015). Although both instrumental and emotional support are linked to higher caregiver well-being in the general population, emotional support has often emerged as a stronger predictor (Cui et al., 2022; Morelli et al., 2015); there may be a differential role for unique kinds of support. Considering the varied needs, potential sources of family support, and the differential impacts on family functioning, a multidimensional framework is needed to capture important nuances in family support for caregivers of autistic individuals and people with DDs.

In autism and DD research, most studies of family support have focused on childhood (Benson, 2012; Bromley et al., 2004; Ekas et al., 2010; McIntyre & Brown, 2018; Robinson & Weiss, 2020) and have indicated that formal support is particularly important to caregivers of autistic children and youth with other DDs (Herman & Thompson, 1995; Vohra et al., 2014). For example, in one study on family support in early childhood, caregivers rated formal sources of support as the most often used (e.g., professions such as a family doctor or professional helpers) and among the most helpful (e.g., school and professional helpers) (McIntyre & Brown, 2018). Prior cross-sectional work found that availability of formal supports for individuals on the autism spectrum or with other DDs and their families decreased with increasing age (Turcotte et al., 2016). Caregivers of adolescents and adults reported increasing difficulty finding sufficient and appropriate formal supports for their autistic loved ones in the years surrounding the transition to adulthood (Anderson & Butt, 2018; Anderson et al., 2018; Rehm et al., 2012). The transition from the school system into adulthood, sometimes termed the “service cliff” (Shattuck et al., 2012), is a vulnerable period when many youths with autism or DD and their families experience considerable changes in—and frequently, losses of—supports and services (Howlin et al., 2005; Taylor & Seltzer, 2011). In the present paper, we provide a unique longitudinal perspective that considers changes in the accessibility, value, and utility of family supports as people with autism or DD and their caregivers age.

Not all family support may be equally helpful or important to caregivers as they and their loved ones with autism or DDs grow older. Preliminary quantitative research in adulthood suggested informal (as opposed to formal) family support played a larger role for caregivers during their child’s adulthood in both families of autistic (Marsack & Samuel, 2017; Marsack-Topolewski, 2020) and non-autistic individuals (Shiba et al., 2016). Although parents of autistic adults reported using some formal sources of support (e.g., approximately 25% used support groups and 50% used psychiatric services), the preponderance of caregivers of autistic adults (88%) reported using informal supports most frequently (Marsack-Topolewski, 2020). Furthermore, among caregivers of aging adults in the general population, informal, but not formal, support was found to impact caregivers’ quality of life (Marsack & Samuel, 2017) and their stress, burden, and depressive symptoms (Smith et al., 2012a). Despite the potential greater reliance on and value of informal supports as caregivers and their autistic children age, recent research suggested that the social networks (i.e., a type of informal support) of caregivers of autistic adults shrank over time (Marsack & Perry, 2018; Yoong & Koritsas, 2012). Thus, longitudinal studies are needed to track fine-grained changes in support across developmental transitions.

This longitudinal study from adolescence to young adulthood used a mixed-methods approach to *quantitatively* examine trajectories of helpfulness and utility of formal and informal family support as well as predictors of those trajectories, including adolescent characteristics (e.g., autism severity, cognitive ability) and family demographics (e.g., race, parent education) in a sample of caregivers of people with autism or DDs. We hypothesized that formal and informal family support would decline across time. Given the limited research in this area, predictors of trajectories were exploratory. We then *qualitatively* assessed change and stability in the sources and characteristics of family support most valued by caregivers for individuals with differing cognitive abilities. There were no a priori hypotheses for qualitative analyses, in light of limited existing research.

## Method

### Participants

The current study included a subset of 126 caregivers enrolled in an ongoing longitudinal study of individuals with autism and related developmental conditions and their family members. Participants were recruited from community-based developmental clinics in three U.S. geographic regions: North Carolina, the greater Chicago area, and Michigan. Caregivers in North Carolina and Chicago were recruited when their child was between ages 2 and 3, and caregivers in Michigan were recruited when their child was approximately age 13. Families from all three sites were followed at the same intervals throughout the study.

To be included in these analyses, caregivers needed to complete the Modified Family Support Scale (Modified-FSS; Bromley et al., 2004) at least once (*M* = 3.5, range = 1–5). In the current subsample, 16.7% of caregivers were Black and 27.0% of caregivers had less than a 4-year college degree. Additionally, 65.6% of all participants lived at home by the end of the study, at approximately age 22 (65.0% of autistic participants; 64.5% of participants without autism). Compared to the longitudinal cohort as a whole, caregivers included in this subset were significantly more likely to identify as White (*p* = 0.002) and to have completed a 4-year college degree (*p* < 0.001); thus, race and education were both tested as predictors in our analyses (see analytic plan below)*.* Caregivers recruited from the Michigan site were also significantly more likely to be included in this subsample (*p* < 0.001); site was accounted for in our multilevel analytic framework. Given that the first instance of Modified-FSS data collection occurred shortly after Michigan caregivers joined the study, lower rates of attrition were expected from this site. Additionally, despite early developmental delays, 23% of the sample never received a formal autism diagnosis throughout the course of the longitudinal study. These participants were retained in analyses due to similar patterns in presentation and outcome across development to the autistic participants (Lord et al., 2020; McCauley et al., 2020). To account for potential diagnostic differences, autism diagnosis and level of autism features (ADOS-CSS, described below) were tested as predictors in multilevel models (see analytic plan). Additional demographic information about this subsample and the full longitudinal cohort is detailed in Table 1.

**Table 1:** Demographic characteristics of sample

	FSS Subsample		Total Sample		*X*^*2*^ (df, n)	*p*
	*n* = 126		*n* = 253			
	ASD	DD	ASD	DD		
	*n* = 97	*n* = 29	*n* = 198	*n* = 55		
Gender						
Male	85	16	159	40	0.83 (1, 253)	0.360
Female	12	13	36	15		
Race						
White/Caucasian	76	27	137	44	12.60 (2, 253)	< 0.001
Black/African American	21	2	60	11		
Recruitment site						
North Carolina	43	10	105	25	18.83 (2, 253)	< 0.001
Illinois	40	1	73	10		
Michigan	14	18	19	20		
Caregiver education						
Graduate or professional degree	26	10	41	8	27.84 (3, 253)	< 0.001
Four-year college degree	37	6	48	7		
Associate degree/some college	20	7	52	11		
High school diploma/GED	14	6	31	14		
Diagnosis						
ASD	97	0	198	0	0.32 (1, 253)	0.567
Non-spectrum	0	29	0	55		

### Procedures

Institutional Review Boards from multiple universities approved this research across the duration of the longitudinal study. Caregivers and, when applicable, participants, provided written consent prior to each assessment. A battery of diagnostic, social-emotional, and psychosocial instruments were collected through in-person visits and mailed questionnaires. In-person assessments occurred multiple times throughout the longitudinal study (ages 2, 3, 5, 9, 18, 21, 26), and questionnaires were completed biannually. Clinicians conducting the in-person assessments were research reliable in the relevant measures and were blind to the participants’ previous assessment results. Diagnoses of autism spectrum disorder (ASD) and/or other disorders were made by the research team and presented to a panel of experienced clinicians for diagnostic confirmation. All assessments were provided free of charge and included feedback.

### Measures

#### Family Support

The Family Support Scale (FSS; Dunst, 1984) was previously adapted for caregivers of children on the autism spectrum (Modified-FSS; Bromley et al., 2004); this Modified-FSS was used in the present study to measure the helpfulness and use of formal and informal family support for caregivers of autistic individuals over time. The Modified-FSS includes 18 items, each indicating a potential source of social support. Each item is rated on two dimensions: helpfulness and frequency of use. For the helpfulness scale (“How helpful do you find the following forms of social support?”), response options were presented on a Likert-type scale with the following response options: “not available”, “not at all helpful”, “sometimes helpful”, and “very helpful”. Consistent with previous research (McIntyre & Brown, 2018), the helpfulness of each source was dichotomized; a source of support was categorized as “helpful” if it was endorsed as “sometimes helpful” or “very helpful.” Similarly, for frequency of use (“How often do you utilize the following forms of social support?”), Likert-type response options included “every day”, “once per week”, “once per month”, and “very rarely or never”; items coded as “used” if the source was used at least once a month. Also consistent with previous autism research (Bromley et al., 2004; McIntyre & Brown, 2018), subscales used in the current analyses included two Informal Support subscales (Family [e.g., partner, relatives, own children, etc.] and Other [e.g., friends, parent groups, co-workers, etc.]) and one Formal Support subscale (e.g., professional support, general practitioners, etc.). Based on theoretical similarity, we consolidated two items on each of the Informal subscales after data collection to ensure the same number of potential sources of support for each of three types of support. More specifically, the higher value between the two support items (within a given subscale) was used for consolidated items. Helpfulness and use of each domain (the dichotomized items) of resources were then summed. Test–retest reliability indicated positive correlations across time with moderate to large effects for Helpfulness (Informal—Family: 0.44–0.71; Informal—Other: 0.52–0.64; Formal 0.38–0.64) and small to large effects for Use (Informal—Family: 0.23–0.67; Informal—Other: 0.09–0.45; Formal: 0.12–0.58). Caregivers also responded to several open-ended questions in writing. For the present qualitative analyses, we focused on the item: “Which of the above sources of social support is the most important to you? Why?”.

#### Autism Features

The Autism Diagnostic Observation Schedule-Second Edition (ADOS-2; Lord et al., 2012) was administered at each in-person visit by an experienced clinician masked to previous diagnostic classification. The ADOS-2 total raw scores were converted to Calibrated Severity Scores (CSS; Gotham et al., 2009) ranging from 1 to 10, with higher values indicating more autism features. ADOS-CSS from the nearest previous time point (approximately age 9) were used in the present study and if unavailable, from later years.

#### Verbal Cognitive Abilities

A set of standardized cognitive assessments were used to measure verbal and nonverbal cognitive abilities at each in-person assessment. A developmentally appropriate assessment was selected from the following: Wechsler Abbreviated Scale of Intelligence (WASI; Wechsler, 1999), Wechsler Intelligence Scale for Children (WISC-III; Weschler, 1991), and Differential Abilities Scale (DAS; (Elliott et al., 1990, 2007). Ratio verbal intelligence quotients (VIQ) were calculated from age equivalents when raw scores did not place within standardized score ranges (see Anderson et al., 2014)*.* IQs from the nearest previous time point (approximately age 9) were used in the present study and if unavailable, from later years. Because of its importance in predicting trajectories of various behaviors, VIQ was used to classify participants as more able (MA; i.e., VIQ ≥ 70) or less able (LA; i.e., VIQ < 70) (Anderson et al., 2014; McCauley et al., 2020).

### Data Analytic Plan

#### Descriptive Analyses

All variables were inspected for outliers and normality using histograms and boxplots. Then, descriptive statistics were computed. Paired-sample *t*-tests were used to compare the three types of social support (Informal—Family, Informal—Other, Formal) with respect to helpfulness. Correlations were then conducted to examine the association between the helpfulness and use of the three types of social support.

#### Multilevel Modeling

##### Data Structure

Multilevel models using maximum likelihood estimation with robust standard errors (MLR) in M*plus* v. 8.1 were built using the following nesting structure: age/time (level 1), individual (level 2), and recruitment site (level 3). First, models were used to test whether between-participant and between-recruitment site nesting accurately captured the structure of the present data by examining the intraclass correlations (ICC; Finch & Bolin, 2017; Luke, 2019). Separate models were constructed for each of our six outcomes; outcomes included helpfulness and use of Informal—Family, Formal, Informal—Other support.

##### Trajectories

Second, unconditional growth models tested the rate of change (i.e., slope) of family support as a function of participant age as a fixed effect. Intercept of the trajectory was set to age 16 (mean age at the first data collection) for ease of interpretation. Third, random effects of age (i.e., differences in slope across people) were added to the model, and improved model fit was tested using a chi-square difference test based on loglikelihood values and scaling correction factors. As such, the model with age as a level 1 predictor addresses our aim to characterize change in family supports over time.

##### Predictors of Trajectories

Fourth, demographic (i.e., race, caregiver education) and individual factors (i.e., VIQ, autism features, autism diagnosis) were each tested individually as predictors of the intercept or slope (i.e., cross-level interactions with age) to determine whether these factors related to initial levels or changes in family supports over time.

#### Qualitative Analysis of Open-Ended Questions

##### Analysis Procedures

Qualitative content analysis (Elo & Kyngäs, 2008) was used to characterize responses to the question “Which of the above sources of social support is the most important to you? Why?”, including the *source* of support and *characteristics* of support. In particular, we used an iterative approach with the following steps: (1) preparing for coding by becoming familiar with the data, (2) generating initial categories, (3) searching for hierarchical structure of categories and subcategories, (4) revising and defining codes and categories, and (5) reporting results (Elo & Kyngäs, 2008). Analyses were conducted primarily from an inductive approach (i.e., identifying codes directly from the data). Notably, aspects of the organizational structure of codes (i.e., categories and subcategories) were well-aligned with theory from previous literature and therefore interpreted as such. For Steps 1 and 2, four authors reviewed the data independently, and each spontaneously generated a list of initial codes. These lists were compared and consolidated collaboratively to form a preliminary list of codes. Then, the same four authors independently searched for a hierarchical structure (i.e., categories and subcategories) among the codes (Step 3), and subsequently met to discuss and come to a consensus and define an initial comprehensive list of categories and subcategories (Step 4). In order to ensure consistency across coders, 20% of the data were coded by all coders and reliability was calculated (all above 80%). During reliability testing, issues with the coding system were identified (e.g., overlapping codes) and resolved collaboratively. Discrepancies were discussed, and a consensus was reached to ensure consistent understanding across coders. The remainder of the open-ended data was double-coded, and discrepancies between coders were resolved through discussion. Coders then discussed responses that did not fit within the existing list of codes, generated new codes, and revised the list of codes, subcategories, and categories as necessary (Step 4). Then, for *source* of support, the percentage of caregivers who endorsed each support source at each data collection timepoint was calculated. Similarly, for *characteristics* of support, the percentage of caregivers who endorsed each category, subcategory, and code were calculated at each data collection time point. Finally, as exploratory analyses, chi-square tests were used to determine whether caregivers who endorsed certain source of support (e.g., formal vs informal) tended to describe particular characteristics of support (e.g., emotional or instrumental). Below, we summarize trends both within and across time as well as differences between caregivers of MA and LA individuals.

##### Positionality Statement

It is important to acknowledge the positionality of the qualitative coders for this research, as such factors may influence the analysis and interpretation of qualitative data. All four coders acknowledge their position as highly educated women in academia. The first author (HS) who organized qualitative coding for this project and led consensus meetings, identifies as an upper-middle class White woman. Of the three additional coders who participated in consensus meetings and subsequent analysis of the qualitative data, one (EC) identifies as an upper-middle class Caucasian woman and the older sibling of a young adult with autism and comorbid intellectual disability (ID), another (NR) identifies as an upper-middle class Caucasian woman, and the third (SG) identifies as an upper-middle class Colombian woman. Although the coders are not caregivers of autistic or DD individuals, the coding team possesses extensive expertise on this topic as the result of clinical experiences and training, interactions with research participants, and personal circumstances. This expertise was important for interpreting this research, and the authors recognize that their backgrounds and prior experiences may have influenced qualitative coding and interpretation in subtle but meaningful ways. That is, everyone holds implicit biases based on their background and prior experiences, which may have influenced how authors code qualitative responses.

## Results

### Descriptive Analyses

All variable distributions were found to be within normal limits, and no extreme values were identified (based on Box plots; extreme values defined as 3 box-length/interquartile range). Highly similar patterns of *t*-test and correlation results emerged at all time points; only the first time point is presented here for sake of parsimony. At time point one (approximately age 16), ratings of helpfulness and use of family supports followed similar patterns such that Informal—Family Support was rated highest, followed by Formal Support, and lastly, Informal—Other Support (Table 2); all comparisons were significantly different (*p* < 0.05). Correlations revealed that level of helpfulness and use were positively associated within each type of support (Table 2). That is, endorsement of sources of support as more helpful was related to more frequent use of that same source of support.

**Table 2:** Correlations among Helpfulness and Use of Support in Adolescence

Variable	*M*	*SD*	1	2	3	4	5
1. Informal—Family—H	3.21	1.21	–				
2. Informal—Other—H	2.17	1.44	0.40**	–			
3. Formal—H	2.85	1.23	0.07	0.40**	–		
4. Informal—Family—U	2.77	1.30	0.54*	0.34**	0.07	–	
5. Informal—Other—U	1.16	1.13	0.01	0.43**	0.10	0.42**	–
6. Formal—U	1.81	1.22	− 0.04	− 0.02	0.39**	0.28*	0.26*

*H* helpfulness of support, *U* use (i.e., frequency of use)

**p < 0.01; *p < 0.05

### Multilevel Modeling

#### Data Structure

The first set of multilevel models was used to determine the appropriate nesting structure of the data. Variation in the helpfulness and use of all family support types (Informal—Family, Formal, Informal—Other) was attributable to between-person differences (ICCs = 0.30–0.53; design effects = 1.75–2.25), but not recruitment site (ICCs < 0.01–0.02; design effects = 1.025–1.05). Given the longitudinal nature of the data and the inherent nesting structure of time within person, a two-level model (time within person) was selected as the most appropriate.

#### Trajectories

The second set of models determined that the trajectory of the helpfulness of Formal and Informal—Other Support declined significantly from adolescence to young adulthood, while the helpfulness of Informal—Family Support did not change significantly across time (Table 3). Additionally, the trajectory of use of Informal—Family Support and Formal Support declined significantly across time, while the slope of use of Informal—Other Support was not significant. Although the random effect of age did not significantly improve model fit for most support subscales, with the exception of Informal—Other Support, it was retained in subsequent multilevel models in order to test cross-level interactions with our predictors of interest (Heisig & Schaeffer, 2019).

**Table 3:** Trajectories of family support from adolescence to young adulthood

	Informal support				Formal support	
	Family		Other			
	Intercept	Slope	Intercept	Slope	Intercept	Slope
Helpfulness of support	3.45**	− 0.04	2.37**	− 0.05*	2.91**	− 0.09**
Use of support	2.97**	− 0.09**	1.28**	− 0.04	1.91**	− 0.08**

Intercept is centered at age 16

**p < 0.01; *p < 0.05

#### Predictors of Trajectories

For helpfulness of all types of support (Informal—Family, Formal, Informal—Other), individual factors (i.e., VIQ, autism features, autism diagnosis) were not significantly related to the level or trajectory of support over time. Of the demographic factors, only caregiver education was significantly related to the intercept of helpfulness of Informal—Other Support (*B* = − 0.49, *p* < 0.001). Higher parental education was related to fewer helpful sources within the Informal—Other Support category.

In contrast, for use of supports, individual factors of the autistic youths predicted trajectories. In particular, autism features were related to the intercept and slope of use of Informal—Family Support (Intercept: *B* = 0.10, *p* = 0.01; Slope: *B* = − 0.02, *p* = 0.02); more autism features were related to greater initial use of family support and greater declines in use across time. Autism diagnosis was related to the intercept of Informal—Other Support (*B* = − 0.41, *p* = 0.04); those who had been diagnosed with autism at some time throughout the longitudinal study demonstrated lower use of Informal—Other Support. VIQ was negatively related to the intercept of use of Formal Support (*B* = − 0.01, *p* = 0.03), such that those with lower VIQs demonstrated more use of Formal Supports. Regarding demographic predictors, only race was related to the slope of Informal—Other Support (*B* = 0.24, *p* = 0.01); Black families demonstrated less decline in use of Informal—Other Supports over time.

### Qualitative Analyses of Open-Ended Questions

#### Sources of Highly Valued Support

Sources of highly valued support were classified using similar categories to our quantitative analyses, including two types of Informal Support (Family and Other) and one type of Formal Support (Table 4).

**Table 4:** Percentage of caregivers who endorsed highly valued sources of support

Sources of support	Time point 1 Age *M* = 16.19, *SD* = 1.25			Time point 2 Age *M* = 17.81, *SD* = 1.30			Time point 3 Age *M* = 18.84, *SD* = 1.34			Time point 4 Age *M* = 19.85, *SD* = 1.28			Time point 5 Age *M* = 21.66, *SD* = 1.25		
	All n = 86	LA n = 50	MA n = 28	All n = 87	LA n = 53	MA n = 27	All n = 86	LA n = 51	MA n = 31	All n = 69	LA n = 43	MA n = 20	All n = 57	LA n = 37	MA n = 15
	%	%	%	%	%	%	%	%		%	%		%	%	%
Family—Informal															
Parents (grandparents)	13.95	14.00	17.86	14.94	18.87	11.11	18.60	25.49	9.68	11.59	11.63	10.00	12.28	16.22	6.67
Sibling i.e., aunt/uncle	5.81	8.00	3.57	4.60	5.66	3.70	3.49	3.92	3.23	2.90	4.65	0.00	5.26	2.70	13.33
Relatives	12.79	10.00	21.43	3.45	15.09	25.93	19.77	17.65	25.81	11.59	6.98	25.00	10.53	10.81	13.33
Partner	40.70	34.00	53.57	39.08	33.96	48.15	41.86	37.25	48.39	37.68	34.88	50.00	40.35	43.24	26.67
Own children (sibling)	13.95	12.00	14.29	14.94	15.09	14.81	12.79	17.65	6.45	13.04	16.28	10.00	15.79	21.62	0.00
Other—Informal															
Friends	13.95	16.00	14.29	11.49	11.32	14.81	19.77	15.69	25.81	11.59	6.98	25.00	14.04	13.51	13.33
Other parents of autistic children	1.16	2.00	3.57	2.30	1.89	3.70	0.00	0.00	0.00	2.90	2.33	0.00	3.51	2.70	0 0.00
Co-workers	2.33	2.00	3.57	2.30	1.89	3.70	1.16	1.96	0.00	4.35	6.98	0.00	1.75	2.70	0 0.00
Social Groups	3.49	4.00	3.57	2.30	1.89	3.70	1.16	1.96	0.00	2.90	4.65	0.00	0.00	0.00	0 0.0
Formal															
Professionals	17.55	20.00	14.29	9.20	11.32	7.41	13.95	11.76	16.13	11.59	9.30	20.00	12.28	16.22	6.67
Educational resources	3.49	2.00	7.14	0.00	0.00	0.00	3.49	0.00	9.68	2.90	2.33	5.00	0.00	0.00	0.00
Respite	1.16	2.00	0.00	2.30	3.77	0.00	1.16	1.96	0.00	0.00	0.00	0.00	0.00	0.00	0.00
Religious organization	5.81	6.00	7.14	10.34	7.55	18.52	12.79	15.69	9.68	5.80	4.65	10.00	7.02	10.81	0.00
School	19.77	22.00	14.29	9.20	9.43	7.41	12.79	17.65	3.23	23.19	27.91	20.00	5.26	8.11	0.00
Government support	0.00	0.00	0.00	1.15	0.00	3.70	1.16	1.96	0.00	0.00	0.00	0.00	0.00	0.00	0.00
Group home	0.00	0.00	0.00	0.00	0.00	0.00	0.00	0.00	0.00	1.45	2.33	0.00	1.75	2.70	0.00
Other															
No support needed	2.33	0.00	7.14	3.45	1.89	3.70	0.00	0.00	0.00	2.90	0.00	10.00	7.02	0.00	26.67
No support available	2.33	2.00	3.57	3.45	5.66	0.00	3.49	1.96	6.45	5.80	6.98	0.00	8.77	5.41	6.67

Missing or un-codable data varied from 14 to 23%; sample sizes and percentages reported above are excluding missing and un-codable data

*MA* more able, *LA* less able

##### Family Support (Informal) Sources

Informal support from family emerged as the most frequently endorsed source of highly valued support (Table 4). Caregivers’ *partners* were endorsed as the most frequently valued source of informal support (ranging from 38 to 42%) across all time points, regardless of the autistic person’s ability level (i.e., MA or LA), as well as the most highly valued source of support overall. Additionally, caregivers consistently described their *parents* (ranging from 11 to 18%)*, extended family* (ranging from 3 to 20%)*,* and *siblings* (ranging from 3 to 6%) as important sources of informal family support. Notably, caregivers’ endorsement of their *other children* who did not have autism (in other words, typically developing siblings of their autistic son or daughter) as a source of informal family support varied across time and ability of the autistic family member (i.e., MA or LA). Specifically, caregivers of MA and LA individuals endorsed *other children* at similar rates (12% and 14%, respectively) at the first data collection time point when caregivers’ autistic children were approximately age 16; however, caregivers of MA autistic individuals reported *other children* as a source of informal support less frequently over time than caregivers of LA individuals. By the last data collection time point, when the autistic individuals were approximately age 22, no caregivers of MA individuals endorsed their *other children* as a source of support. In contrast, caregivers of LA individuals consistently reported their *other children* as a valued informal support over time, with 21% of caregivers of LA individuals describing their other children as an important source of informal support.

##### Other Informal Support Sources

Informal—Other Supports were endorsed as highly valued less often than both Informal—Family Support and Formal Support sources, with relative consistency across time and ability (Table 4). *Friends* were the most commonly endorsed Informal—Other source of highly valued support (ranging from 12 to 20%). This pattern was consistent across time and youth ability. Caregivers also reported *other parents of children with autism, co-workers*, and *child social outlets* (e.g., school clubs, after-school programs, tutoring programs) as other informal sources of support (ranging from 0 to 4%), though less frequently than *friends*.

##### Formal Support Sources

Formal sources of support were endorsed as highly valued more often than Informal—Other sources but less often than Informal—Family Support across time (Table 4). A decline in reported value of Formal Support was observed in young adulthood (Table 4), with notable differences between ability groups. Specifically, while caregivers of LA participants continued to endorse Formal Supports as highly valued into adulthood, caregivers of MA participants reported a steep decline in the value of Formal Supports with age. By the last time point (approximately age 22), over half of the sources of Formal Support continued to be endorsed as highly valued by caregivers of LA participants, while only a single caregiver of an MA participant endorsed any Formal Support, in this case *professional support*, at that time. Caregivers of LA participants described various sources of formal support, including *professional support* (e.g., case workers, psychologist, general practitioner, etc.), *educational resources* (e.g., skills workshops, parent groups, etc.), *school*, *respite care*, *religious organizations*, *state/federal policies* (e.g., Medicare, US Army, etc.), and *group home* services. *School* and *professional support* emerged as the most commonly endorsed sources of Formal Support (ranging from 6–24 to 10–18%, respectively).

#### Characteristics of Highly Valued Support

Overall, two overarching categories emerged related to characteristics of highly valued support (Table 5). First, the type of support (i.e., what the support source is doing to provide support), and second, support person factors (i.e., particular features of that source of support). These two categories were further divided into various subcategories which consisted of multiple codes (Table 5). Similar categories, subcategories, and codes emerged across all five time points, although frequency of endorsement varied. Across all codes, the most frequently endorsed characteristics of valued sources of support included being *dependable*, *understanding*, and providing *partnership* (each discussed in more detail below); these top characteristics were relatively consistent across time and ability group (i.e., MA and LA).

**Table 5:** Percentage of caregivers who endorsed characteristics of valued support sources

Categories, subcategories, & codes	Time point 1 Age *M* = 16.19, *SD* = 1.25			Time point 2 Age *M* = 17.81, *SD* = 1.30			Time point 3 Age *M* = 18.84, *SD* = 1.34			Time point 4 Age *M* = 19.85, *SD* = 1.28			Time point 5 Age *M* = 21.66, *SD* = 1.25		
	All n = 67	LA n = 39	MA n = 25	All n = 63	LA n = 40	MA n = 19	All n = 52	LA n = 29	MA n = 19	All n = 51	LA n = 33	MA n = 15	All n = 39	LA n = 23	MA n = 15
	%	%	%	%	%	%	%	%	%	%	%	%	%	%	%
*Category: type of support*															
Subcategory: emotional support															
Partnership	8.96	7.69	12.00	11.11	12.50	10.53	17.31*	10.34	26.32	17.65*	21.21*	13.33	23.08*	30.43*	6.67
Belongingness	0.00	0.00	0.00	4.76	7.50	0.00	1.92	3.45	0.00	1.96	0.00	6.67	0.00	0.00	0.00
Spirituality	0.00	0.00	0.00	1.59	2.50	0.00	1.92	3.45	0.00	1.96	3.03	0.00	2.56	4.35	0.00
Listening	7.46	7.69	8.00	3.17	0.00	10.53	11.54	10.34	5.26	7.84	3.03	13.33	12.82	13.04	13.33
Elicits positive emotions	0.00	0.00	0.00	4.76	2.50	10.53	5.77	10.34	0.00	3.92	3.03	6.67	2.56	4.35	0.00
Love and/or caring	4.48	5.13	4.00	1.59	0.00	5.26	9.62	10.34	10.53	3.92	6.06	0.00	7.69	8.70	6.67
Subcategory: instrumental support															
Provides intervention or education to autistic person	10.45	7.69	16.00*	4.76	5.00	5.26	7.69	10.34	0.00	15.69*	9.09	33.33*	5.13	8.70	0.00
Provides education and training to parent	2.99	2.56	4.00	1.59	0.00	5.26	1.92	0.00	5.26	1.96	0.00	6.67	0.00	0.00	0.00
Helps with planning for future	1.49	2.56	0.00	4.76	7.50	0.00	0.00	0.00	0.00	1.96	0.00	6.67	0.00	0.00	0.00
Provides respite/childcare	16.42*	25.64*	4.00	9.52	12.50	5.26	3.85	6.90	0.00	9.80	15.15	0.00	7.69	8.70	0.00
Provides help with problem solving	7.46	5.13	8.00	4.76	2.50	10.53	15.38	6.90	31.58*	3.92	6.06	0.00	2.56	4.35	0.00
Provides help with daily tasks	7.46	7.69	8.00	4.76	2.50	5.26	1.92	3.45	0.00	9.80	9.09	6.67	2.56	0.00	6.67
Provides structure and activities for child	5.97	7.69	0.00	0.00	0.00	0.00	0.00	0.00	0.00	1.96	3.03	0.00	7.69	8.70	0.00
*Category: support person factors*															
Subcategory: knowledge, values, & characteristics															
Has understanding or acceptance of child	14.93*	23.08*	4.00	11.11	7.50	21.05*	9.62	13.79	5.26	15.69*	12.12	20.00*	12.82	13.04	6.67
Has expertise in autism or child development	4.48	0.00	12.00	1.59	2.50	0.00	0.00	0.00	0.00	1.96	3.03	0.00	2.56	4.35	0.00
Has shared values, perspective, beliefs	1.49	0.00	4.00	1.59	2.50	0.00	0.00	0.00	0.00	1.96	0.00	6.67	2.56	0.00	6.67
Has positive personal characteristics	0.00	0.00	0.00	0.00	0.00	0.00	0.00	0.00	0.00	1.96	0.00	6.67	5.13	4.35	6.67
Has understanding of parents experiences	7.46	7.69	8.00	14.29	12.50	15.79	11.54	6.90	10.53	9.80	9.09	6.67	12.82	13.04	6.67
Subcategory: willingness to help															
Offers support before being asked	2.99	5.13	0.00	4.76	7.50	0.00	3.85	3.45	5.26	3.92	3.03	0.00	0.00	0.00	0.00
Dependable and/or available/accessible	11.94	7.69	20.00*	28.57*	35.00*	15.79*	30.77*	31.03*	31.58*	11.76	9.09	13.33	25.64*	30.43*	20.00*
Subcategory: logistics															
Duration of relationship	0.00	0.00	0.00	1.59	0.00	5.26	1.92	0.00	5.26	0.00	0.00	0.00	0.00	0.00	0.00
Has frequent contact with child or caregiver	5.97	7.69	4.00	15.87*	17.50*	15.79	11.54	17.24*	5.26	11.76	18.18*	0.00	5.13	4.35	6.67
*Category: other*															
Subcategory: no support															
No supports needed	2.99	0.00	8.00	6.35	2.50	10.53	0.00	0.00	0.00	3.92	0.00	13.33	10.26	0.00	26.67*
No support available	1.49	0.00	4.00	4.76	7.50	0.00	7.69	3.45	10.53	7.84	9.09	0.00	12.82	8.70	6.67

*MA* more Able, *LA* less able

*Top two most frequent codes at that time point; missing or un-codable data varied from 34 to 47%; sample sizes and percentages reported above are excluding missing and un-codable data

##### Category: Type of Support

Many caregivers described ways in which support was provided, which were characterized as emotional (e.g., *partnership*, *caring*) or instrumental (e.g., providing *childcare*, helping with *planning for the future*, helping with *daily tasks*) (Table 5).

##### Subcategory: Emotional Support

Emotional support was defined as eliciting positive emotions or making someone feel loved and cared for by providing empathy, kindness, a listening ear, and/or feelings of belongingness and solidarity. Emotional support as a characteristic of valued support demonstrated an increasing pattern across the study, suggesting an increase in the relative importance and value of emotional support across the transition to adulthood (Table 5). This pattern was particularly pronounced for caregivers of LA individuals. Within the specific emotional support codes, providing a sense of *partnership* was most commonly endorsed, both across time and ability group. As noted above, *partnership* was also one of the most common codes overall (within and across time and ability group). For example, caregivers valued *partnership* “because we have a common bond, reference, and investment,” “because he is my team wholeheartedly,” and “because we feel we are in this together, partners.” Other emotional support codes included *belongingness* (e.g., “trying to help him be a part of the community”), *listening* (e.g., “will always listen to my concerns” and “it’s always been helpful to me to be able to just talk”)*, eliciting positive emotions* (e.g., “makes me feel good about myself” and “source of happiness”), *meets a need for spirituality* (e.g., “all things work together for good to them that love the Lord”), *and love and/or caring* (e.g., “unconditional love”, “genuinely care”, and “we love each other”).

##### Subcategory: Instrumental Support

Instrumental support includes help with practical aspects of caregiving, including respite or childcare for the caregiver as well as assistance with practical decision-making (e.g., financial planning). On average, endorsement of instrumental support as a valued support characteristic declined over time (Table 5) in both ability groups, indicating decreased availability and/or importance of instrumental supports with increasing child age. However, the most common instrumental support codes differed across ability groups. Compared to caregivers of MA individuals, caregivers of LA individuals were more likely to highlight providing respite/childcare as a key component of instrumental support. Caregivers noted multiple sources of respite/childcare support (e.g., “trained behavioral staff allows [my son] to remain at home” and “my daughter is my best support, she allows me some respite time”). For caregivers of MA individuals, the most commonly endorsed instrumental support codes were *helps with planning for the future* (e.g., “vocational rehabilitation, because they are helping him with career planning”), *provides intervention, services, or education to child* (e.g., “[they are] helping him with school work and exam prep”), and *help with problem-solving and decision making* (e.g., “they get down to the real ‘nitty gritty’ with money and time”), although the frequency of endorsement varied across time.

##### Category: Support Person Factors

In addition to describing the particulars of the type of support, caregivers also described features of the support person(s), including their knowledge, values, and characteristics (e.g., having expertise in autism), their willingness to help (e.g., availability), and logistical aspects related to the support (e.g., frequency of contact) (Table 5).

##### Subcategory: Knowledge, Values, & Characteristics

Overall, the knowledge, values, and characteristics of the support source demonstrated relatively similar patterns for caregivers of MA and LA groups (Table 5). Examination of specific codes revealed that *having understanding/familiarity and/or acceptance of the child* or *having an understanding of parents’ experiences* were the most commonly reported characteristics across ability groups. For example, caregivers said, “because they understand my son,” “they understand what I am going through,” and “[they] know and understand challenges.” Other codes within this subcategory included *expertise in autism/child development* (e.g., “knows condition”)*, shared values/perspective/beliefs* (e.g., “we share similar parenting styles”)*,* and *positive characteristics of the person* (e.g., “is patient and tolerant”), although these were endorsed less frequently.

##### Subcategory: Willingness to Help

The support source’s willingness to help emerged as a highly valued feature of the support person, especially when the autistic youths were around age 18 (time points 2 and 3) (Table 5). This pattern was driven primarily by caregivers in the LA group. Willingness to help included codes such as the *dependable* nature of the support person and the support person’s *proactive offering of support*, regardless of the form of support and the intended recipient of the support (i.e., directed at helping the caregiver, autistic individual, family, etc.). Upon analyzing specific codes, being *dependable* was identified as the most important characteristic of the support person both within the “willingness to help” subcategory and across ability groups. The *dependable* code captured responses from caregivers including “they will always be there for me”, “they are available daily to support [my child] in all areas of her life”, “reliable and enjoys helping”, and “have support when and if we need it”. The other code within this subcategory included *proactive offering of support*, which, although infrequently endorsed, represented responses such as “they volunteer and ask me if they can help—I don’t feel like I’m imposing on them.”

##### Subcategory: Logistics

Caregivers, a majority of whom were from the LA group, also referenced logistical aspects of support when describing why certain support sources were most valued (Table 5). Examination of specific codes revealed that *having frequent contact* was the most commonly endorsed logistical characteristic. For example, caregivers reported “everyday access,” “watch [the child] every Friday night,” and “help daily.” The other code within this subcategory was the *duration of the relationship*, which captured responses including “I’ve been with her for years” and “has stayed involved with [the child] for 18 years”.

#### Links Between Sources & Characteristics of Highly Valued Support

Chi square tests revealed that formality of support source was significantly related to type of support at ages 20 and 22, and marginally related at age 18. In particular, at ages 18 and 22, a larger proportion of caregivers endorsed instrumental support from formal support sources (approximately 73 to 75% of formal support sources) than from informal support sources (12 to 30% of informal support sources). At ages 20 and 22, a larger proportion of caregivers endorsed emotional support from informal sources (60 to 82% of informal support sources) than from either formal support sources (0% of formal sources at age 20) or the combination of both formal and informal sources (0% of caregivers who endorsed both formal and informal sources at age 22).

Formality of support was significantly related to person factors only at age 18. A greater proportion of caregivers described willingness to help from informal sources (50% of informal sources) than formal sources (0% of formal sources).

## Discussion

These findings provide, to our knowledge, the first mixed-methods longitudinal characterization of family support across the transition to adulthood among families of autistic youth and individuals with other developmental delays. Previous research has highlighted the clear impact of family support on both caregiver and youth functioning in autism and DDs (e.g., Ekas et al., 2010; Vasilopoulou & Nisbet, 2016), yet existing literature has primarily focused on childhood using cross-sectional studies, which are limited by cohort effects, particularly into adulthood. As such, this study adds to the growing body of research by following families longitudinally during the transition to adulthood. Quantitative analyses revealed declines in formal support and certain aspects of informal family support from adolescence into young adulthood. Differences in the use, but not helpfulness, of support emerged based on youth/young adult autism features, cognitive ability, and other demographic factors (e.g., maternal education). Qualitative analyses further shed light on these experiences by highlighting families, especially partners, as a highly valued source of support, with shifts in the kinds of support valued across the years. At times, unique patterns were evident for caregivers of MA and LA adults; for example, support from their other children was more highly valued by caregivers of LA, compared to MA, individuals over time. Overall, these findings suggest that the observed cross-sectional shift from formal to informal support from childhood through adulthood in autism is primarily driven by a decline in formal support, as opposed to an increase in informal support.

Based on quantitative and qualitative analyses, support from family was rated as the most helpful (quantitative), used (quantitative), and valued (qualitative) source of support in our sample. Additionally, although use of family support declined over time on average, especially for youths/young adults with more autism features, the helpfulness and value of family support remained relatively constant. From a social convoy framework (Antonucci et al., 2014), families often occupy the inner-most space of social networks, leading to a combination of physical and emotional closeness that can be leveraged over time. For example, spouses not only play a key role in terms of “tag-team” parenting (Hock et al., 2012), meaning they often share care responsibilities, but they can also offer a sense of partnership, as reflected in our qualitative analyses. This finding has important implications for these families, given the high rates of divorce among couples raising an autistic child (Berg et al., 2016; Hartley et al., 2010), particularly among caregivers of MA individuals in adolescence (Bahri et al., 2022).

Other support from family comes from the caregivers’ parents and their other children. Grandparents play an important support role and have been found to often provide both monetary and caregiving support for children with DDs or autism (Harper et al., 2013; Hillman et al., 2016; Prendeville & Kinsella, 2019). Nevertheless, barriers can complicate a grandparent’s ability to provide support (e.g., living far from their family, financial burden) (Hillman et al., 2017). While support from grandparents, especially in the form of respite/childcare, can alleviate parenting stress (Harper et al., 2013), aging grandparents may be less able to provide support over time due to normative aging challenges. Finally, other children in the family (i.e., siblings of autistic child or child with DDs) also often provide caregiving support for their autistic sibling or sibling with DDs both during childhood and later in life (Lee & Burke, 2018; Nuttall et al., 2018; Robinson et al., 2016). Given the lifelong duration of the sibling relationship (Cicirelli, 1982), siblings may transition into the primary caregiving role as parents age. Thus, siblings represent an important source of support for caregivers of individuals with autism and DDs (Heller & Arnold, 2010; Lee & Burke, 2018; Tozer et al., 2013). Collectively, our results highlight the importance of support from close family members during the transition to adulthood in this population.

Compared to both support from family members and formal sources, supports from other informal sources (e.g., friends) were reported to be less frequently used (quantitative), helpful (quantitative), and valued (qualitative), with use being particularly low for families with youths diagnosed with autism. Additionally, helpfulness (but not use or value) of other informal support declined across the transition to adulthood. It may be that the more distal nature of these sources of support, in terms of both physical distance and theoretical proximity, made these supports harder to access (Antonucci et al., 2014). Given that caregiving responsibilities persist into many autistic children’s adulthood (Shattuck et al., 2012), forming and maintaining informal bonds with people such as friends or co-workers is likely a challenge. Notably, caregivers with less education and Black caregivers reported other informal support as initially more helpful and showed less decline in use compared to caregivers with more education and White caregivers, respectively. These findings highlight the need for a better understanding of the contextual, cultural, and societal factors impacting caregiver support (Carr & Lord, 2013; Kim et al., 2020).

Additionally, the only diagnostic difference (i.e., between participants diagnosed with ASD vs. participants diagnosed with other DDs) in family supports was that caregivers of individuals with DDs were more likely to use informal sources of support than caregivers of autistic individuals. This difference may be due to differing opportunities for informal connections. For example, non-autistic people may have more friends due to no or lessened presence of social challenges, and by extension, caregivers may have a larger social network with other parents.

Families of youths with lower cognitive ability, and thus greater support needs, reported greater use of formal support in adolescence based on quantitative analyses. However, declines in the helpfulness (quantitative), value (qualitative), and use (quantitative) of formal sources of family support occurred, on average, across all participants in the study. The declines in formal support are consistent with the “service cliff” that many autistic adults experience during the transition to adulthood (Shattuck et al., 2012, 2020). In the United States, approximately 50,000 autistic individuals turn eighteen each year (Roux et al., 2013). As these individuals exit school in their late teens and early twenties, they lose access to an array of school-based services—including occupational and speech therapy, social supports, and life skills training (Henninger & Taylor, 2013; Wei et al., 2014). Although the receipt of these services may end abruptly during the transition to adulthood, the service needs of young adults with autism or DDs and their caregivers continue well into adulthood and beyond. A growing body of work highlights the many challenges autistic individuals face in early adulthood, including high rates of depressive and anxious symptoms (Hollocks et al., 2019), slowing or declines in adaptive skills and symptom improvement (Clarke et al., 2021; Smith et al., 2012b; Taylor & Seltzer, 2010), and struggles to achieve normative outcomes (McCauley et al., 2020). Due to these challenges and the lack of services in adulthood, qualitative (Anderson & Butt, 2018) and quantitative (Marsack & Samuel, 2017; Turcotte et al., 2016) investigations of caregiver well-being and experiences indicate that more support is needed for caregivers as their children move into adulthood.

Qualitative content analyses helped characterize highly valued support sources according to the ways in which support was provided (e.g., instrumental or emotional) and features of the support source (e.g., their knowledge of autism). Regarding type of support, we saw an overall shift from valuing instrumental support to emotional support across the transition to adulthood, especially for caregivers of LA adults. This shift is likely not due to changes in residential status or independent living, as 65.6%, or almost two-thirds, of our sample lived at home throughout the study. These changes over time may reflect the changing needs of caregivers as they and their children age. It is also possible that the declining availability of formal supports (e.g., service cliff) and in-home supports influenced these findings, as caregivers may have only been important aspects of support they were actively receiving; thus, further examination is warranted. These qualitative findings regarding emotional and instrumental support are consistent with our quantitative results that indicated a decline in formal support and relative stability in aspects of informal support. Based on these similarities in patterns and our exploratory analyses, it seems to be that informal support tends to be more emotional in nature while formal support tends to be more instrumental, although this is not always the case. Additionally, fine-grained analyses revealed the importance of a sense of partnership and dependability for caregivers throughout their children’s adolescence and young adulthood across both time and ability group. Caregivers valued feeling like they had someone alongside them, navigating together their child’s transition to adulthood and feeling as though they could turn to someone for support if needed. These results speak to the value of simply “being there” for caregivers of MA and LA adults alike.

With the exception of use of informal supports, we are struck by the considerable similarities between caregivers of autistic individuals and caregivers of individuals with other DDs in our findings. These similarities mirror other findings showing similar trajectories of caregiver well-being (Singer et al., 2023) and adult outcomes (e.g., employment, living independently, and having friends and romantic relationships) (Chan et al., 2018; Lord et al., 2020). The challenges faced by individuals with autism and other DDs and their caregivers appear to be more similar than different, and future work should continue to examine similarities and differences in these related but distinct groups.

### Limitations and Future Directions

Despite the many strengths of this study, including the longitudinal design and mixed-method approach, these findings should be considered within the context of several limitations. In particular, this sample is composed of a unique group of caregivers of individuals with autism or DDs. These caregivers sought help in the first years of their child’s development during the early 1990s, an era in which formal services were much less available than they are today. Thus, the participants in this sample may be meaningfully different from caregivers of individuals diagnosed later in development or diagnosed very early in life today. In addition, attrition has impacted the number of Black caregivers and caregivers with less than a 4-year college degree in this study. Finally, we have relatively few women in this sample, which constrained our ability to test for differences by gender.

Furthermore, although this study begins to fill a gap in the literature by examining social support beyond childhood, future research should explore how support changes into later adulthood. We need to better understand the consequences of social support changes for adults with autism or DDs and their families as they age. Additionally, though this study was able to tease apart different sources of support (i.e., informal vs. formal), other support dimensions may be important to the well-being of caregivers and their loved ones with autism and should be explored in future studies. For example, support can be either perceived (i.e., support believed to be available should an event occur) or enacted/received (i.e., support actually provided during stressful events; Birditt et al., 2012); these dimensions have been found to only modestly correlate (Haber et al., 2007). Future studies should also evaluate whether the identified family support needs and preferences are consistent across both mothers and fathers, given that gender-based caregiving norms may necessitate different types of support. For example, mothers have been shown to desire a greater network of informal supports than fathers to ameliorate stress levels from direct contact with daily caregiving as their autistic children age (Tehee et al., 2009).

### Implications

Given the importance of support for well-being and other outcomes of caregivers (e.g., Ekas et al., 2010; Vasilopoulou & Nisbet, 2016), the identified declines in the helpfulness, use, and value of support, particularly formal support, highlight the need to bolster the accessibility of family support during the transition to adulthood for families of autistic and DD youth. Support for caregivers is not often included as part of best practice guidelines in providing services to autistic or DD people, especially after early childhood (e.g., Amer et al., 2022; Coury et al., 2020; Sanchack, 2020). Therefore, we encourage service providers across medicine, education, and social services to ask about and consider caregiver support needs and resources, including informal supports, into their child’s adulthood. It may be helpful to integrate family support as a part of transition plans (e.g., identifying from who and how the family will be supported as the student leaves school). Furthermore, considering the strengths of support from family members (i.e., reported as the most helpful, most used consistently across time, and most valued), promoting support amongst family members may be an important target of intervention. Based on our qualitative analyses, clinicians may want to prioritize different types of support (e.g., instrumental or emotional) depending needs of the family and current life circumstances. Helping families identify and form connections with at least one person whom they can depend on may make a difference for caregivers navigating their child’s adulthood.

### Conclusion

Across the transition to adulthood, support from family members was reported to be most consistently helpful, used, and valued by caregivers of autistic individuals or those with DDs. In contrast, formal support (e.g., professionals, school) was found to decline over time. Caregivers, especially those of LA adults, reported a shift from strongly valuing instrumental support to valuing emotional support as they and their autistic youth aged. A sense of *partnership* and *dependability* was most critical to caregivers. Overall, these findings fit within a social convoy perspective and likely reflect the “service cliff” experienced by people with autism or DD as they transition into adulthood. While caregiver social networks shrink over time and formal services are less readily available in adulthood, remaining sources of close-knit support, particularly from family members, become increasingly important.
